# Bird species detection by an observer and an autonomous sound recorder in two different environments: Forest and farmland

**DOI:** 10.1371/journal.pone.0211970

**Published:** 2019-02-07

**Authors:** Kinga Kułaga, Michał Budka

**Affiliations:** Department of Behavioural Ecology, Adam Mickiewicz University in Poznań, Poznań, Poland; University of Lleida, SPAIN

## Abstract

Birds are commonly used as bio-indicators of the quality of environments and the changes to them. Therefore, ecologists put a lot of effort into the monitoring of their population trends. One of the methods used for bird population monitoring is autonomous sound recording. Current studies provide inconsistent results when the number of detected species by autonomous sound recorders was compared with that delivered by an observer. In our study, observers counted birds using a point-count method at 64 random points in forest and farmland. At the same points, autonomous sound recorders recorded the soundscape four separate times (including counting by observer period) and the species present in the recordings were later identified by observers in the lab. We compared the number of species detected by simultaneous observations and recordings, as well as the number of species detected by recorders during four different surveys. Additionally, we calculated the Sorensen index to compare the species composition during different surveys at the same point. We found that observers detected more species than autonomous sound recorders. However, differences in the number of detected species were habitat dependent–observers detected more species than recorders in farmland, but not in the forest. When the time for recording was doubled, recorders were more effective than observers during a single survey. The average Sorensen index between the four repeated surveys performed by autonomous sound recorders ranged from 0.58 to 0.67, however we did not find significant differences in the number of species detected during different surveys conducted at the same point. Our study showed that 10-minutes sampling from the same point gives various species composition estimates but not species richness estimates between different surveys. Therefore, even when recorders detect less species than observers during the simultaneous surveys, increasing the survey duration of recorders may alter this difference. The use of autonomous sound recording for monitoring bird populations should be promoted, especially in forest habitats, as this technique is easier to standardise, eliminates many errors observed in the traditional point-count approach, enables conducting survey during adverse field conditions and delivers more reliable results for the majority of the species.

## Introduction

Changes of population size, density and distribution are some of the most important measures describing how populations of terrestrial animals respond to environmental changes [1]. Therefore, ecologists and conservation biologists put a lot of effort into monitoring population trends of many animal species [2–3]. Population monitoring allows for guidance for the management of populations: being able to measure the effects of protective activities and natural perturbations; documenting compliance with regulatory requirements and detecting incipient disturbance [4].

Through the use of birds as bio-indicators, the results of ornithological monitoring can be used to assess the impact of adverse environmental changes on living organisms and to study their reactions to these changes [5–6]. The occurrence of birds in a wide range of environments and their high biodiversity gives the opportunity to estimate the condition of many different habitats [7]. A characteristic of many bird species is their quick response to environmental changes, therefore allowing for the detection of such changes in a short time frame [8–9].

One of the most widely used techniques for the census and monitoring of bird diversity and abundance is the point–count method [9–10]. The point-count method, with various modifications, has been applied to bird counts in different regions of the world and in various habitats [11–13]. Generally, for point–count methods, observers record all birds seen and heard for a set period of time within a fixed distance around the point [9–10]. Each individual detected is usually assigned to one or more distance bands (e.g.: within 100 m [14]; 0–30 m and over 30 m; or 0–30 m, 30–100 m and over 100 m [15]). The recommended minimum distance between points is 200 m, reducing the probability of counting the same individual at different points [15]. Typically, the standard counting time is 10 minutes [9–11] [16], however, researchers often modify the duration of counting and use four, five, six minutes or a multiple of those times [9–10]. At least two counts in temperate regions are required during the breeding season [17]. Such numerous variations of the point-count method depend on the target of the survey–species, group of species or all species, as well as the specific environmental conditions in which birds are counted. It is important to note that the point–count method delivers results that are an estimation of the real bird species composition, distribution and abundance. However, the point-count method provides a way in which to monitor large areas in a short time.

Independently of the method used, field observers encounter challenges related to the accurate assessment of the position and distance to an observed bird [17–20]; the different detection probabilities of different species [21–22]; the various activities of birds during different times of the day or season [23] as well as the influence of the observer on a birds behavior [24]. Therefore, new methods of bird monitoring that are universally applicable and more effective, whilst at the same time eliminating present counting errors and delivering more reliable results, are still being discussed.

Detectability is usually variable, both between- and within-species, depending on many factors. Such differences in detectability influence the accuracy of estimations both for species composition and density [18]. For example, blackbirds (*Turdus merula*) begin to sing long before sunrise, as one of the first birds in the dawn chorus, with the next peak of vocal activity being observed in the evening [25]. Alternatively, tawny owls (*Strix aluco*) are active all night, from dusk to dawn, but during the day are extremely secretive and virtually impossible to detect acoustically [26]. Thus, the detection probability for these two species (and many others) is different if we look at a single point within the whole day. Additionally, some bird species, such as the wood pigeon (*Columba palumbus*), common chiffchaff (*Phylloscopus collybita*) or skylark (*Alauda arvensis*) are easy to detect and count in a single control period, whereas other species like the european nightjar (*Caprimulgus europaeus*), eurasian eagle-owl (*Bubo bubo*) or middle spotted woodpecker (*Dendrocopos medius*) are much more difficult to detect in a single control [27]. This is because of their latent lifestyle and so observers are only able to record a low percentage of present individuals within a study plot [28].

Detection probability is also modified by unfavorable weather conditions. Strong wind, fog or rain reduces acoustic detection and visibility of birds, therefore, particular individuals are more difficult to detect in such conditions [18]. Such weather may also reduce bird activity, another factor contributing to a lower detectability of individuals [9] [29]. However, even when observations are conducted during optimal weather, some bird species may vary in detectability depending on the habitat type in which the survey is conducted [30]. In wooded habitats, vegetation is dense, and so the detection of birds is usually due to the recognition of songs and calls as visual detection is difficult [31]. In contrast, visibility in open areas is very good, therefore, observers are able to see the whole area in which birds are counted, meaning most species can be detected visually, even when they are silent [32].

Another factor influencing the probability of detection are the individual skills and experience of the observer [32–34]. Most large–scale monitoring programs use volunteers with a diverse set of skills and experience, changing the accuracy of bird detection. Even long–term monitoring projects that are conducted by the same observers may generate systematic errors. As observers acquire more experience their ability increases and therefore they should detect birds more effectively. Some longitudinal research, such as the 40 year monitoring scheme for birds in Białowieża National Park, require skill verification for a potential observer, helping to reduce the variability in the probability of birds being detected between observers and years [35]. An additional factor affecting bird detectability is the presence and behaviour of an observer in the field, as this may cause a change to birds normal behaviour [24] [36]. Some of them may stop or even dramatically change their singing behaviour whilst others may choose to leave an area around the point or transect. A consequence of such a change in behaviour may be that birds cannot be detected during a counting period, or some may be more easily detected but are being misrepresented due to the abnormal increase in singing behaviour.

The point-count method uses bird locations, which may distort the accuracy of birds monitoring. This problem is associated with the estimation of the distance to birds from the observer, and may generate significant errors, even when the position of an individual bird is classified into one of two distance categories: more than 100 m or less than 100 m from observer [29] [34]. Thus, errors in distance estimation can provide incorrect information of the bird population structure in the study area [20]. However, this problem may be minimised by thorough training of observers [31].

Human based counting approaches, and some of the errors related with these methods, could be eliminated by autonomous sound recordings [37–40]. Autonomous sound recording is a widely used sampling tool in ecological research and population monitoring [39]. In autonomous sound recording, bird songs and calls are recorded using autonomous sound recorders at a single point for many hours. These recorders have numerous benefits for ecological research including: the ease of repeated sampling across spatial and temporal scales; a reduction in observer bias; a reduction in the time spent in the field by qualified ornithologists [40]. This method should enable the detection of all vocally active birds in a radius around the recording point, however, silent species can only be detected visually. Moreover, the vocal activity of birds is species-specific and changes both seasonally and daily [41–42]. Autonomous sound recorders may follow these activity changes through the correct set up of the start time and end time of a recording. Alternatively, they may record continuously for many hours or days. Another challenge of autonomous sound recording is related to the differences in song amplitudes amongst species, and as a consequence, the various distances from which songs of different species can be recorded by autonomous sound recorders [40] [43]. Therefore, it is difficult to compare abundance and distribution of different bird species based on autonomous sound recorders, since various species are recorded from different distances. However, it was recently shown that it is possible to standardise the detection range and estimate distance to singing individuals from autonomous sound recordings [44]. Overall, autonomous sound recording seems to be a promising method in bird detection and surveying, but detailed tests are needed to estimate the potential errors in detection of particular species as well as the development of standardised survey and analysis protocols [45]. Across numerous studies, comparisons of detectability of birds by autonomous sound recorders and field observers do not report consistent results. Some studies suggest autonomous sound recording is better, others favour traditional observer methods, whilst some studies suggest there is no difference between these two methods [40] [43] [46].

In this study we compared the number of species detected through human observers (in the field) and recordings by autonomous sound recorders (manually analysed by observers in the lab), at the same time, within two different habitats–forest and farmland–in the temperate region of central Europe. We expected that habitat type may significantly influence species detection by the observer, therefore differences in the number of species detected by observer and autonomous sound recorder may differ between habitats. We also identified which species are detected visually, acoustically, or visually and acoustically by the observer. Additionally, we examined whether the species composition at a point is constant when surveys are conducted at different times of the day, or across different days, by an autonomous sound recorder. We also examined how increasing the number of surveys for autonomous sound recorders may improve species detection.

## Methods

### Study site and points selection

The study was conducted in Upper Nurzec River Valley in Eastern Poland (52°36′N, 23°14′E). This typical farmland landscape is comprised of two main habitat types. The first is a large area of peatland meadows, situated in wide depressions within the terrain that stretches along the Nurzec river. The second habitat type is extensively farmed arable fields (mainly winter and spring cereals, occasionally corn and root crops) located on the edge of valley. Agriculture is rather extensive in the study area (small field area, diversity of crops, traditional farming, balks, low fertilisation), and so consequently, over 140 breeding bird species have been observed here, including typical farmland bird species like the skylark, yellowhammer (*Emberiza citronella*), whinchat (*Saxicola rubetra*) with rare and endangered species like the great snipe (*Gallinago media*), black grouse (*Tetrao tetrix*) and black-tailed godwit (*Limosa limosa*) [47].

Approximately 700 ha (17% of the entire area) of the Upper Nurzec River Valley is covered by forests. More than half of the woodlands are coniferous forest, with the remaining area being covered by mixed forests and in a lesser degree, deciduous forests. Woodlands form small dismembered complexes, with the exception of the large complex (above 100 ha) located in the north-eastern part of the valley. In the forests of the Upper Nurzec River Valley species such as the european pied flycatcher (*Ficedula hypoleuca*), dunnock (*Prunella modularis*) and hazel grouse (*Tetrastes bonasia*) are known to breed [46].

We randomly selected 64 counting points within the study area based on a satellite image. 32 points were located in the meadows and arable fields, whilst the other 32 points were in forest habitat (the distance from the point to the forest edge was higher than 100 m). The distance between each counting point was greater than 500 m, thus minimalizing the probability of recording the same individuals at different points. The average distance between the nearest points was 558 m.

### Counting methods

At each point an autonomous sound recorder (Song Meter SM3 Wildlife Acoustics with two built-in omnidirectional microphones; signal-to-noise ratio 68 dB) continuously collected data from one hour before sunrise until 09:00 the next day. We used 16 different recorders. Depending on the condition of the habitat we placed recorders on trees or shrubs between 2 and 5 m above ground level. On the first day the recorder was set up. On the next day the human observer approached the recording point and counted birds for a 10 minutes period using the standard point-count method. The counting of birds by an observer took place from 4:17 to 7:57 (local time). Observers counted all birds and noted the type of species detection: visual, acoustic or visual and acoustic. Field observations were noted by using Olympus LS-12 digital recorder (instead of paper field sheet). The sound recording session and observer-based surveying took place during the breeding season from April 29 to May 6, 2018. Point counts were performed by two field observers (KK and MB), with various experience in counting birds (3 vs 15 years; less vs more experienced observer). Counting points were randomly assigned to each field observer. The weather condition during the fieldwork was appropriate to conduct the research as there was no strong wind or rain.

### Bioacoustics analyses

The autonomous sound recorders collected two-channels recordings (separate channel for each microphone) with a 48 kHz/16 bit sampling rate. Bioacoustics data were analysed using Avisoft SAS Lab Pro.5.2.12 software. Two observers (KK and MB) used 1024 FFT Length, 75% Frame and Hamming Window to view the spectrograms. During manual scanning of the spectrograms and listening of recordings, the observer classified all recorded songs and calls to a particular species and noted the time (in seconds) at which the first song of a particular species was detected during the 10-minutes recording session. When observers had difficulty with species recognition they compared unrecognized songs with bird song samples available on-line through the bioacoustics library of the Xeno-Canto Foundation [48]. Each observer counted birds at 32 points and analyzed recordings collected by autonomous sound recorders at the remaining 32 points in which they did not count birds. In this way we ensured that previous presence of an observer in the point did not influence species detection in the lab. To examine whether observers differ in the number of species detected in the lab, we randomly selected 20 recordings analyzed by one observer and re-analyzed them independently by the second observer. We found no significant differences in the number of detected species by two observers (paired t-test: t = -1.453; df = 19; p = 0.163).

**Fig 1:**
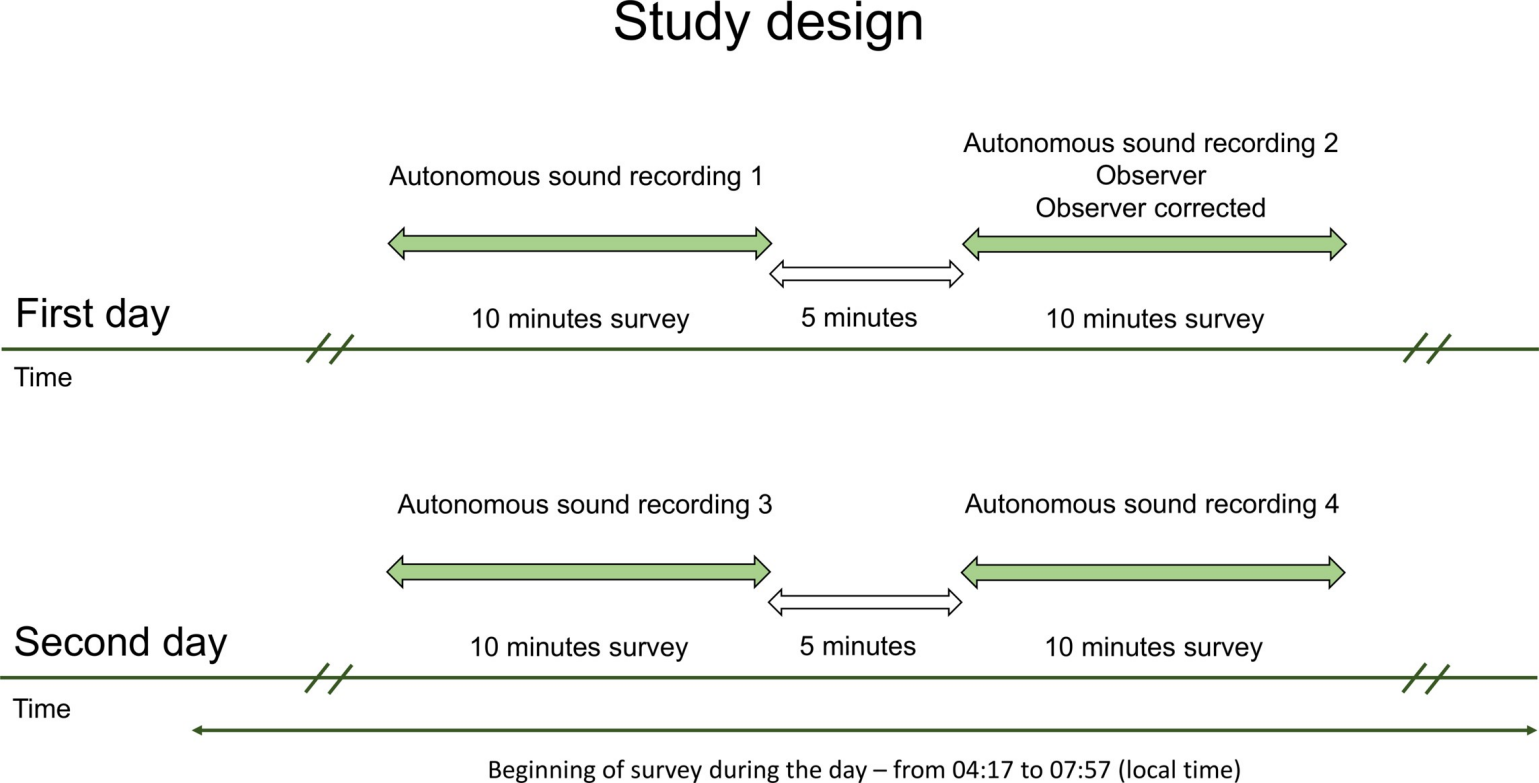
Temporal study design. Number of species detected during a single survey was based on: (1) autonomous sound recording (four surveys per point), (2) counting by observer, (3) corrected counting by observer (autonomous sound recording + observer counting in the same time).

For each counting point, four (10-minutes each) recording sessions recorded by the autonomous sound recorder were analysed: (1) 15 minutes before the observer had started counting, (2) at the same time at which observers counted the birds, (3) at the same time as number 1 but the next day, (4) at the same time as number 2 but the next day (Fig 1). For the analyses we used two sets of data collected by observers in the field during a 10-minute counting period. The first set contained results of a typical point-count (all species heard and seen by the observer in the field). The second set contained the cumulative number of species detected by observer and recorded by autonomous sound recorder surveying birds in the same time (corrected observer counting). We assumed here that observers had suitable hearing abilities and were able to detect all vocally active species. Using two sets of observer-based data in our analyses we addressed two different issues: (1) a simple comparison of field observer data with autonomous sound recorder data and (2) a comparison of corrected field observer data with autonomous sound recorder data, which showed the problem of a various and usually unknown observers' skills, and the consequences of it on interpretation of the studies comparing detection of birds by field observers with autonomous sound recorders.

Finally, we collected data from 64 points. For 54 points we conducted five surveys: (1) observer, and autonomous sound recorder recording: (2) at the same time as the observer counted birds, (3) 15 minutes before the observer had started counting, (4) at the same time as counting by the observer but a day after, (5) 15 minutes before the observer had started counting but a day after. Due to technical problems, on eight points we collected data from three surveys (1–3) and on two points data from one survey (only counting by observer).

### Statistics

Firstly, we calculated the number of species detected during the study, the frequency of a particular species detected during a single survey (number of surveys in which species was detected / number of all surveys) and the frequency of the species at points at which five surveys were conducted (number of points in which species were detected / 54 points in which five surveys were conducted). We also determined which species were only recorded in the forests, in the open areas and in both types of habitat. Next we determined in which way (visually, acoustically or visually and acoustically) each bird species was detected by the observer.

The number of detected species by different methods at the same time and at the same point was compared using a Generalised Linear Mixed Model (GLMM), with point ID defined as a subject and various survey methods as repeated measurements. We compared the number of species detected by an observer, corrected field observer and autonomous sound recorder performed at the same time. In the model we included survey type (observer, corrected field observer and autonomous sound recorder), observer (less vs more experienced), type of habitat (forest vs farmland) and two-way interactions between those three variables as fixed effects. Data were fitted using a negative binomial distribution with a log link function. An analogous model (but without interactions between fixed effects) was conducted to examine differences in the number of species detected by autonomous sound recorders during four surveys conducted in two succeeding days at the same point.

In the next GLMM model, we examined how increasing the number and time of a survey for autonomous sound recorders improved species detection. We used the same set of fixed effects as the previous model (without interactions between fixed effects).

We used the Sorensen index (QS) to compare similarity of species composition between different surveys at the same point. To calculate the Sorensen index we used the following formula $QS\;=\frac{2\times C}{A+B}$, where A and B are the numbers of species in the two different samples, with C being the number of species shared by the two different samples. The calculation of the Sorensen index provides information about the similarity of the species compositions we detected in the same point during different surveys. The index ranges from 0 to 1, where 0 is no similarity in the groups (no shared species) and 1 is groups that have exactly the same species composition (the same species are observed in group A and B). All statistical analyses were done in IBM SPSS 24 software. R software version 3.4.2 [49] was used to calculate Sorensen index [38]. All p-values are two tailed.

### Ethics statement

In our study we did not conduct experiments with animals, therefore we did not need any special permissions. Access to our study area is not restricted in any way. According with Polish law, access to public and private-owners’ lands (including forests and agricultural areas) is not restricted, excluding fence areas or areas where is no entry. Most of the survey points were located on public areas, we did not meet lands to which accessibility was limited or illicit. No specific permissions were required for conducting our study. The study did not involve endangered or protected species.

## Results

A total of 86 species were detected during the study. The frequencies of particular species were diverse (S1 Table). The most frequently observed species during a single survey were the common cuckoo (*Cuculus canorus*) (61% of surveys), skylark (55% of surveys) and common wood pigeon (46% of surveys), while the common linnet (*Linaria cannabina*), mute swan (*Cygnus olor*) and grey heron (*Ardea cinerea*) were only recorded occasionally (observed during 0.3% of surveys). The most widespread species for the 54 points in which five surveys were completed was the common cuckoo. There were 18 species that were only detected at one point during the whole study period (e.g.: common linnet, eurasian bullfinch (*Pyrrhula pyrrhula*) or green sandpiper (*Tringa ochropus*)). We found 26 species which were observed only at points located in the forests, 27 species which were observed only at points located in farmland and 33 species observed in both types of habitat (S1 Table).

### Species detection during surveying by an observer

Human observers conducted 64 surveys at 64 points, and detected 73 bird species altogether. 12 species were only detected visually, 20 only acoustically and 41 both visually and acoustically. From the 612 observations in which species were identified by observers in the field, 65% were identified acoustically, 15% visually, whilst 20% both visually and acoustically (S2 Table). When we looked at the proportion of the various types of species identification in forest and farmland we found significant differences between these two habitats (χ2 = 72.68, df = 2, p < 0.001). In the forest 82% of species identification were done acoustically, 14% acoustically and visually and 4% visually. Whilst in the farmland 51% were identified to a species acoustically, 26% acoustically and visually and 23% visually.

### Number of species detected by simultaneously conducted methods

**Fig 2:**
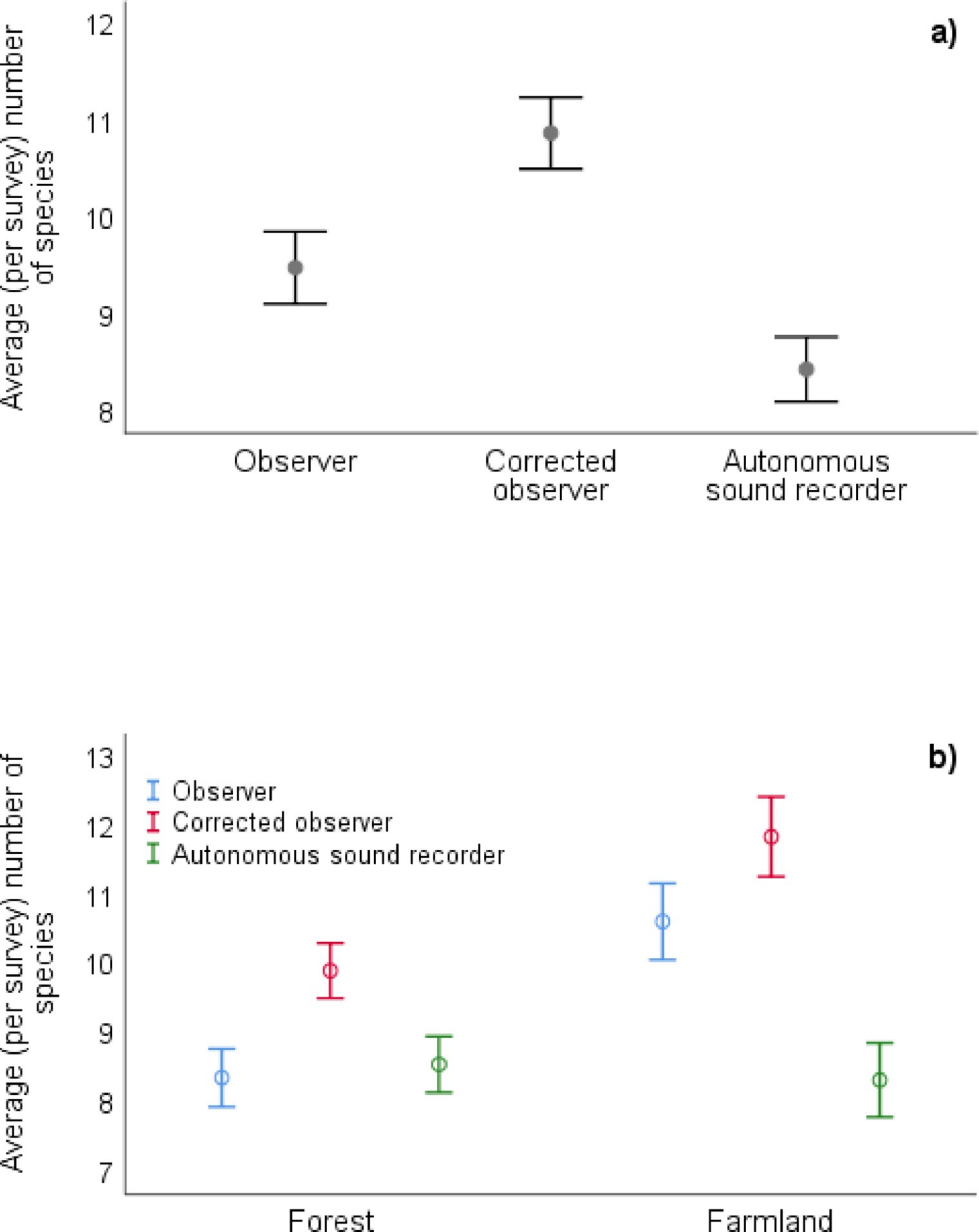
Influence of survey method, habitat type and experience of observer on number of detected species. Graphs show: **a)** An average (per survey) number of species detected in various types of survey: counting by observer, corrected field observer (observer + autonomous sound recorder) and autonomous sound recorder; **b)** An average (per survey) number of species detected in different types of habitat (forest and farmland) in various types of survey. Bars represent ± standard error of mean.

In the 62 points in which surveys were conducted simultaneously by observer and autonomous sound recorder, observers detected 73 species compared to autonomous sound recorders that detected 63 species (S3 Table). A GLMM model showed that the autonomous sound recorder detected significantly less species than the observer and corrected field observer (Fig 2A and Table 1). However, the correlation between the number of species detected by an observer and the autonomous sound recorder was high and significant (Pearson correlation: r = 0.631, p < 0.001, n = 62). The interaction between habitat and survey type showed a habitat-dependent pattern in species detection by various survey methods. A significant difference in the number of species detected by an observer and autonomous sound recorder was observed in the farmland but not in the forest (Table 1 and Fig 2B).

**Table 1:** Results of GLMM, showing the influence of factors on the number of detected species by various survey methods conducted at the same time.

	Coefficient	SE	t	p
**Intercept**	**2.024**	**0.075**	**26.845**	**<0.001**
Habitat:				
Forest	-0.045	0.088	-0.511	0.610
Survey:				
** Observer**	**0.507**	**0.085**	**5.995**	**<0.001**
** Corrected observer**	**0.561**	**0.086**	**6.561**	**<0.001**
Observer:				
Less experienced	0.167	0.087	1.916	0.057
Habitat x Survey:				
** Forest x Observer**	**-0.233**	**0.097**	**-2.392**	**0.018**
** Forest x Observer corrected**	**-0.193**	**0.098**	**-1.975**	**0.050**
Habitat x Observer:				
** Forest x Less experienced**	**0.171**	**0.073**	**2.331**	**0.021**
Survey x Observer:				
** Observer x Less experienced**	**-0.590**	**0.098**	**-6.032**	**<0.001**
** Corrected observer x Less experienced**	**-0.439**	**0.098**	**-4.484**	**<0.001**

Dependent variable: number of detected species; fixed effects: habitat (forest vs farmland), survey (observer, corrected observer, autonomous sound recorder), observer (less vs more experienced), interactions between: habitat and survey, habitat and observer, survey and observer; subject: point ID (unique number of point); repeated measures: various survey methods; data fitted by using a negative binomial distribution with a log link function. Corrected Akaike for the model is 50.868. Significant effects (p < 0.05) are bold.

Regarding the observer and corrected-observer based surveys, the human observer with more field experience detected significantly more species than the less experienced one. However, this was not the case when analysing recordings collected by autonomous sound recorders in the lab (Table 1). When only analysing detections by observers for different habitats, without the influence of survey type, we found that the more experienced observer detected significantly more species than the less experienced one in farmland, but not in the forest (Table 1).

### Number of species detected by autonomous sound recorder during different surveys at the same point

A GLMM model showed no differences in the number of species detected during four different surveys conducted by an autonomous sound recorder at the same point, as well as no differences in the number of species detected in the farmland and forest habitats. The only significant difference was that the less experienced observer detected more species in the lab than the more experienced one (Table 2).

**Table 2:** Results of GLMM, showing the factors influence on the number of detected species by autonomous sound recorder during different surveys at the same point.

	Coefficient	SE	t	p
**Intercept**	**1.961**	**0.050**	**39.498**	**<0.001**
Habitat:				
Forest	0.079	0.042	1.882	0.061
Survey:				
15 minutes before, day after	-0.007	0.059	0.119	0.905
Day after	-0.061	0.058	-1.053	0.293
15 minutes before	0.074	0.056	1.309	0.192
Observer:				
** Less experienced**	**0.248**	**0.042**	**5.888**	**<0.001**

Dependent variable: number of detected species; fixed effects: habitat (forest vs farmland), survey (autonomous sound recording during counting by observer, 15 minutes before counting by observer, and analogous two surveys next day), observer (more vs less experienced); subject: point ID (unique number of point); repeated measures: various survey methods; data fitted using a negative binomial distribution with a log link function. Corrected Akaike for the model is 158.504. Significant effects (p < 0.05) are bold.

### Influence of increasing the number and duration of the survey on the number of species detected

**Fig 3:**
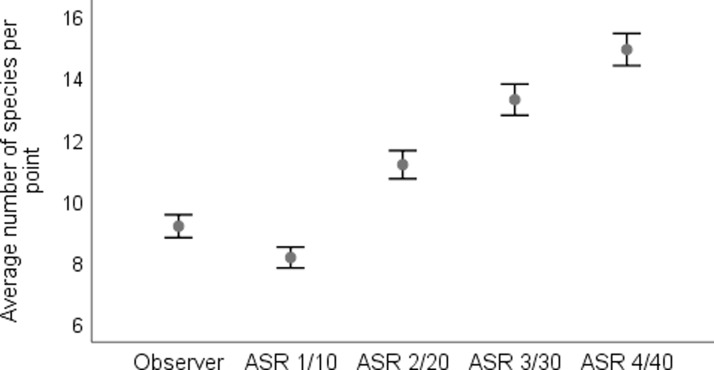
The cumulative average number of species (± SE) detected per point during the different number and duration of surveys. The average number of species detected by an observer and the cumulative number of species recorded by autonomous sound recorder are given. ASR 1/10, ASR 2/20, ASR 3/30, ASR 4/40 indicate the number of species detected by the autonomous sound recorder during different number of surveys (1–4) / different cumulative survey duration (10–40 minutes). Surveys one and two were done during the same day, while surveys three and four being completed during the second day.

We examined how increasing the number and duration of autonomous sound recorder surveys increases the number of detected species. A GLMM based on 54 points at which one survey by an observer and four surveys by autonomous sound recorder were completed, showed that increasing the time and number of surveys by two times enabled the detection of significantly more species than during a single survey by an observer. The less experienced observer detected more species in the lab than the more experienced one. Habitat type was not significant in this model (Table 3 and Fig 3). Counting by an observer and the simultaneous recording by an autonomous sound recorder delivered 73 and 62 species, respectively. However, autonomous sound recorders detected 76 species during four surveys.

**Table 3:** Results of the GLMM showing how increasing the number and duration of surveys increases the number of detected species.

	Coefficient	SE	t	p
**Intercept**	**2.102**	**0.056**	**37.319**	**<0.001**
Habitat:				
Forest	0.040	0.033	1.193	0.234
Survey:				
ASR (1 survey / 10 minutes)	-0.177	0.064	-1.847	0.066
** ASR (2 surveys / 20 minutes)**	**0.197**	**0.063**	**3.133**	**0.002**
** ASR (3 surveys / 30 minutes)**	**0.369**	**0.061**	**6.005**	**<0.001**
** ASR (4 surveys / 40 minutes)**	**0.485**	**0.060**	**8.083**	**<0.001**
Observer:				
** Less experienced**	**0.187**	**0.033**	**5.576**	**<0.001**

Dependent variable: number of detected species; fixed effects: habitat (forest vs farmland), survey (autonomous sound recorder–ASR; observer), observer (more vs less experienced); data fitted using a negative binomial distribution with a log link function. Corrected Akaike for the model is 116.576. Significant effects (p < 0.05) are bold.

### Similarity of species composition at point

We used the Sorensen index (QS) to compare the similarity of species composition between different surveys at the same point. When comparing species composition detected by an autonomous recorder and an observer and a corrected observer at the same point and time, we found significant differences (Kruskal Wallis test; χ^2^ = 46.503, df = 2, p < 0.001). The average Sorensen index was the highest (QS = 0.93, MIN = 0.40, MAX = 1.00) when the observer and corrected field observer were compared, lower for a comparison of recorder and corrected observer (QS = 0.85, MIN = 0.71, MAX = 1.00) and the lowest for the recorder and observer comparison (QS = 0.78, MIN = 0.40, MAX = 1.00) (Table 4).

**Table 4:** Similarity of species composition recorded by autonomous sound recorders at the same points in different surveys.

	QS (mean)	N	SD	Min	Max
ASR–ASR DA	0.58	54	0.136	0.14	0.86
ASR–ASR BDA	0.60	54	0.116	0.31	0.83
ASR–ASR B	0.67	62	0.122	0.40	0.88
ASR B–ASR BDA	0.60	54	0.138	0.27	0.86
ASR DA–ASR BDA	0.63	54	0.136	0.23	0.94
ASR B–ASR DA	0.63	54	0.123	0.23	0.92
OBS COR–ASR	0.85	62	0.128	0.40	1.00
OBS-OBS–COR	0.93	62	0.081	0.71	1.00
OBS–ASR	0.78	62	0.140	0.40	1.00

ASR–data from an autonomous sound recorder during counting by an observer; ASR B–data from an autonomous sound recorder 15 minutes before an observer had started counting; ASR DA–data from an autonomous sound recorder at the same time as counting by the observer but a day after; ASR BDA–data from an autonomous sound recorder 15 minutes before an observer had started counting but a day after; OBS–data from observer survey; OBS COR–corrected data from observer survey (observer + autonomous sound recorder).

**Fig 4:**
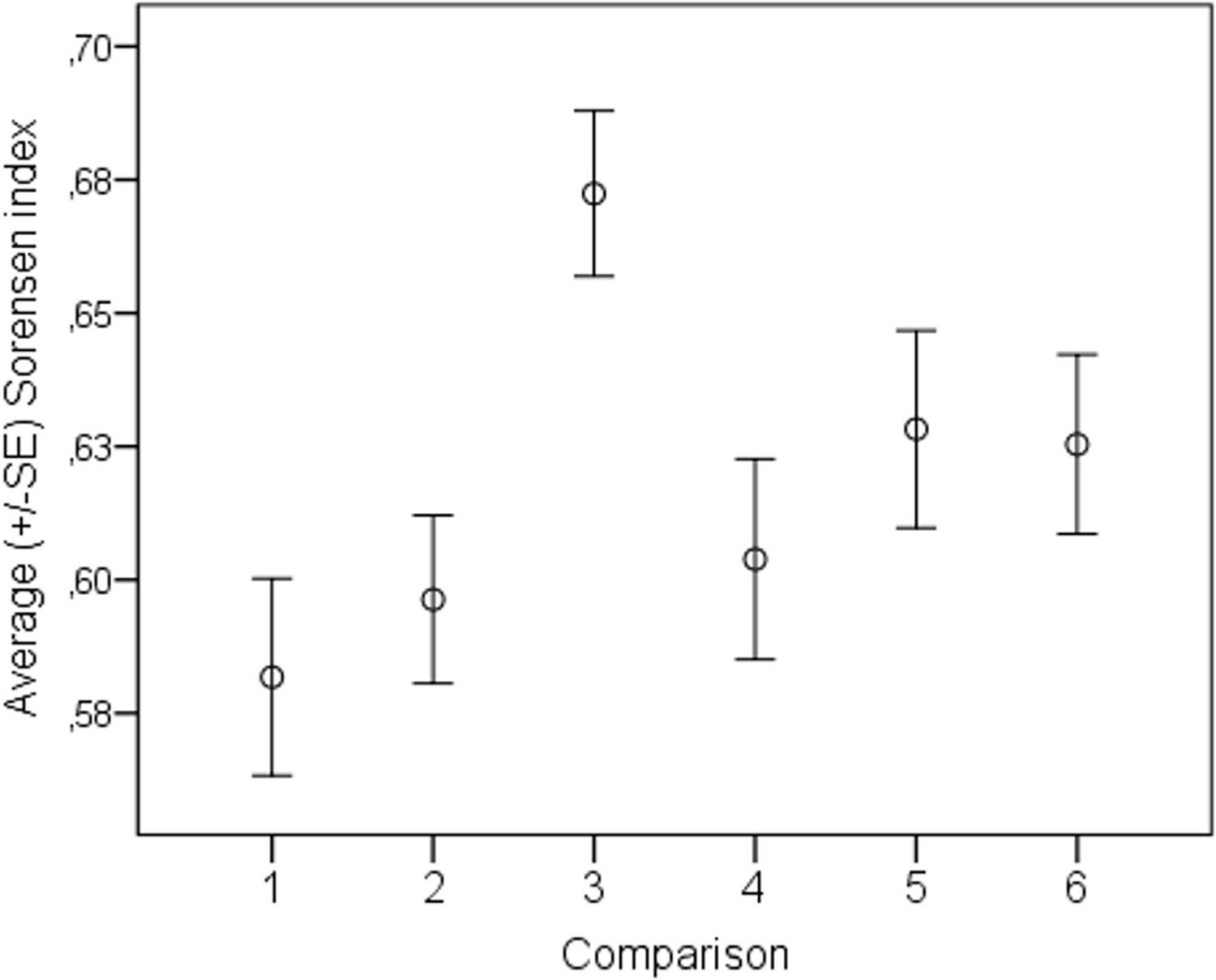
Similarity of species composition recorded by autonomous sound recorders at the same points at different times. 1 –during counting by an observer vs day after in the same time; 2 –during counting by an observer vs day after but 15 minutes earlier; 3 –during counting by an observer vs 15 minutes before an observer had started counting; 4–15 minutes before an observer had started counting vs day after in the same time; 5 –next day during counting by an observer vs the same day but 15 minutes earlier; 6–15 minutes before an observer had started counting vs during counting by an observer but next day.

When we compared bird species composition at the same point using just the autonomous sound recorders during different the four repeated surveys, we found significant differences (Kruskal Wallis test; χ^2^ = 15.749, df = 3, p = 0.008; Fig 4). The average Sorensen index for all of the dataset was 0.62 (N = 332, SD = 0.131, MIN = 0.14, MAX = 0.94; Table 4). The highest value of the Sorensen index was observed when the data from an autonomous sound recorder conducted simultaneously to an observer was compared with data from an autonomous sound recorder survey conducted 15 minutes before an observer had started counting. The lowest value of the Sorensen index was observed when data from an autonomous sound recorder conducted simultaneously to an observer was compared with data from the same scenario but a day after. For more details see Table 4.

## Discussion

Our study showed that the number of species detected by an observer during a 10 minutes survey was significantly higher than the number of species detected by an autonomous sound recorder for the same time. Similar results were reported for birds communities in the rainforests of central Australia [46], boreal forests in Canada [50] and the USA [43]. Such a pattern seems to be expected, since silent species are not possible to detect acoustically, but may be detected visually by human observers. In our study, observers detected 12 species only through visual means (e.g. swallows, birds of prey, waders, storks, herons, ducks, swans; see S2 Table for more details), but such silent, and acoustically undetectable species, have also been noted in others studies [43] [50–53]. Most of these silent species are not necessarily a subject of the breeding birds monitoring project as they are only seen to be feeding around the survey point or have very large territories and do not breed directly around the survey points. Species suited for surveying schemes are those which occupy small- and medium-sized territories for which the point-count method can be utilised. Such species are continuously present around the survey points, using vocalisations to attract mates (frequency is dependent on time of season and day), repel rivals and signal danger [54]. Such continuous vocal activity is why such territorial species should have similar detectability by observers and autonomous sound recorders. It seems that compared with highly qualified observer, autonomous sound recorders may, at most, detect the same number of species as just using an observer, when we assume that detectability distance by recorder and observer is similar [50]. In a few studies, like in the open areas in Brazil (Brazilian Cerrado) [52] or primary montane forests of Mount Cameroon [53], researchers did not find significant differences in the number of species detected by observers and autonomous sound recorders. Therefore, inconsistent results of previous studies [40] may be due to the various skills of observers counting birds, which are obvious in ornithology [33–34] [55]. This is a general problem when comparing the effectiveness of observers and autonomous sound recorders–less experienced observers involved in counting birds will generate results supporting autonomous recorders, while the results of more experienced observers support human observations. Another factor which may influence results of the studies comparing recorders and observers is various quality of equipment used by researchers. Simply, a various-quality equipment cause that birds singing with the same amplitude are recorded from a various and usually unknown distance. In our study we used 16 identical recorders and two observers. To show how many singing species is omitted by observers we took together the number of species detected by an observer and recorded by autonomous sound recorder in the same time. The total number of species detected by these two methods was significantly higher than those detected by the observer or autonomous recorder, meaning that field observers, independent of experience, overlooked vocalizing species. This problem is evident in habitats where most of the species are detected acoustically and where lots of species sing together. In the field the perceptional abilities of the observer changes depending on the number of species heard and seen at the same time, whereas in the lab, it is possible to listen to a recording and scan the spectrogram multiple times [43] [56]. In such cases, the probability of detecting birds is higher when recordings from autonomous sound recorders are analyzed in the laboratory than during a field survey. In our study a less experienced observer detected more species in the lab than a more experienced one. However, despite random assign of points, the less experienced observer analyzed in the lab more species-rich sites, and counted in the field less species-rich sites than the more experienced observer.

We found that when only using visual detections, 4% of detected species were identified in forest and 23% in farmland. This disproportion was also reflected in the effectiveness of autonomous sound recorders in those two habitats. We did not find significant differences in the number of species detected by an observer and autonomous sound recorder in the forest, whilst in the farmland such a difference was significant (Fig 2B). Similar results were reported for two tropical bird species (black throated bobwhite *Colinus nigrogularis* and spot-breasted wren *Pheugopedius maculipectus*) which were better detected by observers in the pastures and coastal dunes but not in forests [57]. On the other hand, Hutto and Strutzman [43] found no significant differences in species detection by observers and autonomous sound recorders among three habitat types: green mixed-conifer forest, burned mixed-conifer forest, and mixed riparian cottonwood bottomland. This suggests that habitat-related differences in species detection between observer and autonomous sound recorder may only occur when extreme habitats are compared (forest vs open area) or when one of the habitats contains more secretive species than the other.

The huge advantage of autonomous sound recorders is that this technique enables for the increase of both the time and frequency of the controls and does not need highly qualified ornithologists in the field. Simply, many recorders may be set up in the field, they may work simultaneously, it the same time in different seasons, what is especially important in temperate regions, where breeding season is relatively short and the number of experienced ornithologist is limited. Many studies show that extending the time of control [34] or appropriate sampling [58] increases the probability of species detection, also true when autonomous sound recorders are applied [59–60]. When we compared the number of species detected by an observer with the number of species detected by an autonomous sound recorder working the same day but twice as long (2 x 10 minutes, 15 minutes before counting and during counting by observer) or during two different days (four times longer; 2 x 10 minutes x 2 days), we found that the autonomous sound recorder was able to detect significantly more species than the observer during a single survey (Table 3 and Fig 3). The difference in the number of species detected by an observer and autonomous sound recorder would be higher if a 24 hour soundscape were to be analyzed for each point, or even when subsamples (10 minutes, one hour) are analyzed from whole-day recording [59]. This way, species active during the evening and night, like owls, crakes, nightjars or woodcocks, would also be detected. Increasing the survey duration or conducting a survey in a very specific, short time of vocal activity (just before dawn or just after dark) was proved to be effective in detecting the european nightjar [61]. Autonomous sound recorders allow for the collection of data at many points at the same time, for many hours or days; eliminates the influence of a field experience observer and, at least in our case, detects more species than an observer when the survey duration by recorder is increased. When all these reasons are regarded, this technique should become the most common method of birds monitoring in the future. A definite advantage is that automatic species recognition is developing and the software for detection is becoming more effective [62–64]. In addition, randomly selecting samples from a whole recording (e.g. one hour or a few 10 minutes samples from 24 hours recording) may be sufficient to detect a high number of the total species present around a recording point [59].

The method of autonomous sound recording does have some limitations which need future study. Different bird species sing at different sound amplitudes [65] and so different species are recorded by autonomous sound recorders from different distances. For example, a calling corncrake male (90–100 dB at 1 m) [66] can be recorded from a further distance than the song of male chaffinch (80 dB at 1 m) [49]. Another problem of autonomous sound recording is related with the variety of environments inhabited by birds. Habitat structure and weather condition significantly influence the sound propagation and degradation of an acoustic signal [51]. What is more, in the same habitat type, sounds with different frequencies propagate and degrade differently [67]. Therefore, the same bird song will be detected by autonomous sound recorder from different distances depending on habitat type, weather condition or background noise. Presently, using autonomous sound recorders for between-species comparisons of distribution or abundance of birds seems to be problematic. At the current stage of knowledge, for the most of the species, we do not have data to estimate distance to singing individual. Simply, measuring, for example, a song amplitude from recording we are not able to say how far is a singing individual, because we do not have data of song amplitude of most of the bird species and decreasing amplitude during song transmission in natural environment. Having said this, field observers also make errors in the judgement of distance to birds in the field [68–69]. Therefore, to avoid these two types of errors in our study, we did not estimate distance to observed birds and considered all the species that were present within the detection abilities of the observer and autonomous sound recorder. The detection space of recorders was unknown in our study, what means that vocalizing species could be detected by recorders from smaller distance than by observer, what supports an observer in detecting species.

Our study showed that the number of species detected by our autonomous sound recorders did not differ significantly between the different 10-minute surveys conducted at the same point (Table 2). In addition, we did not find evidence of the avoidance effect of birds when presented with human observers, at least in the context of species richness (Table 2). However, we highlighted a general problem with the repeatability of species composition delivered by 10-minutes surveys. We use the Sorensen index to compare species composition at the same point during different counting sessions. The highest similarity in species composition was observed when we compared surveys conducted simultaneously by an observer and autonomous sound recorder (Table 4). When we compared all possible combinations of species composition only detected by autonomous sound recorders, we found that the average similarity ranged between 0.58 to 0.67 (Table 4 and Fig 4). We did not find a point at which the same species composition was recorded during two recorder-based surveying sessions (QS_max_ = 0.94). What is more, the highest value of the Sorensen index was observed when the autonomous sound recorder was conducted simultaneously to human observation and the survey 15 minutes before. This means that the avoidance effect in the context of species composition was not observed in our study. Such low similarity in the species composition support the results of earlier studies that suggest that increasing the survey duration by autonomous sound recorder and appropriate sampling may increase species detection, especially those which vocalize rarely or in a very short and specific time of a day [59–60].

In conclusion, in our case, observers were able to detect more species than autonomous sound recorders. However, differences in the number of detected species were habitat dependent–in farmland, observers detected more species than autonomous sound recorders, but not in the forest. When the duration of survey for autonomous sound recorders doubled, recorders were more effective than the human observer during a single survey. The similarity in species composition (Sorensen index) between the observer and autonomous sound recorder working simultaneously (QS = 0.78) was higher than the similarity between two different surveys conducted by autonomous sound recorder at the same point (QS range from 0.58 to 0.67). Therefore, using an autonomous sound recorder for monitoring bird populations seems to be welcomed, since this technique is easier to standardize, eliminates some errors observed in the traditional point-count approach; delivers more reliable results and, when using appropriate sampling and increasing the survey duration, detects more species than an observer during a single count.

## Supporting information

S1 Table Bird species detected at points during the study. The table shows frequency of species during different types of survey: Freq Observer–counting by an observer; Freq Autonomous sound recorder–data from an autonomous sound recorder during counting by an observer; Freq Observer corrected–data from autonomous sound recorder and observer counting birds in the same time; Freq Autonomous sound recorder 15 minutes before–data from an autonomous sound recorder 15 minutes before an observer had started counting; Freq autonomous sound recorder day after–data from an autonomous sound recorder at the same time as counting by the observer but a day after; Freq autonomous sound recorder 15 minutes before day after–data from an autonomous sound recorder 15 minutes before an observer had started counting but a day after), Freq Survey–frequency of the species during single survey; Freq Point–frequency of the species at points on which five surveys were conducted; Freq Forest–frequency of the species in Forest; Freq Farmland–frequency of the species in farmland. All frequency present in per cent.
(XLSX)

S2 Table Species detection during surveying by observers. Type of detection is shown: Visually–birds noted only by visual detection; aurally–birds noted only by aurally detection; Visually and aurally–birds detected both by visual and aurally detection. Table based on 64 counts by observer conducted on 64 points.
(XLSX)

S3 Table Dataset. Species detected during surveys conducted in 64 points. Columns indicate: Point ID–number of point; Coordinates–geographical coordinates; Habitat–habitat type; Survey–type of survey (Observer–counting by observer; Observer corrected–counting by observer and autonomous sound recorder in the same time; ASR–autonomous sound recorder, ASR 15 before—autonomous sound recorder 15 minutes before an observer had started counting; ASR DA–autonomous sound recorder at the same time as counting by the observer but a day after; ASR 15 before DA–autonomous sound recorder 15 minutes before an observer had started counting but a day after); Date–date of survey; Time–hour of survey; Observer–observer who counted birds in the field or analysed recording in the lab; Species (English)–English name of the species; Species (Latin)–Latin name of the species.
(XLSX)
